# Detection of genes mediating beta-lactamase production in isolates of enterobacteria recovered from wild pets in Saudi Arabia

**DOI:** 10.14202/vetworld.2015.1400-1404

**Published:** 2015-12-17

**Authors:** Sabry A. Hassan, Mohammed Y. Shobrak

**Affiliations:** 1 Department of Biology, Faculty of Science, Taif University 888, Taif, Saudi Arabia; 2 Department of Microbiology, Faculty of Veterinary Medicine, South Valley University 83523, Qena, Egypt

**Keywords:** animal exhibit, extended-spectrum β-lactamases/AmpC beta-lactamase, fecal samples, polymerase chain reaction, Saudi Arabia

## Abstract

**Aim:**

To determine the genetic basis and types of beta-lactamase encountered among enterobacterial isolates of wild pets from the animal exhibit.

**Materials and Methods:**

A total of 17 beta-lactamase-producing enterobacteria recovered from fecal samples of wild pet animals were analyzed for a selected beta-lactamase gene by polymerase chain reaction.

**Results:**

Molecular analysis identified one or more β-lactamase-encoding genes in 14 enterobacterial isolates as a single or gene combination. The most frequent extended-spectrum β-lactamases types were TEM and CTX-M, and the most common AmpC enzymes were CMY-2 and DHA types.

**Conclusions:**

The study is the first in Saudi Arabia, have established the presence of β-lactamase-encoding genes in the fecal isolates of wild pets.

## Introduction

Antibiotic-resistant bacteria are extremely important to human health. The production of ß-lactamases is the major mechanism of bacterial resistance to β-lactam antibiotics which are considered the most widely used class of antibiotics against both Gram-negative and Gram-positive bacteria. Resistance to this class of antimicrobial agents is therefore of immense clinical significance.

A major reason for resistance of *Enterobacteriaceae* to beta-lactam antibiotics is the production of extended-spectrum β-lactamases (ESBLs) and AmpC beta-lactamases, capable of inactivating the effects of broad-spectrum cephalosporins and penicillins [1]. Exposure to ESBL/AmpC-producing microorganisms can occur through any means, but the hospital has always been thought to be the greatest risk [2]. The occurrence of ESBL/AmpC-producing microorganisms is on the rise globally, with prevalence varying from country to country and within a country from institution to institution [3]. The genes that encode for these enzymes may be plasmid-borne or chromosomally located.

Wild animals provide a biological mechanism for the spread of antibiotic resistance genes [4]. Recently, a number of studies describing the occurrence of ESBL-resistant *Escherichia coli* in wildlife [5-14].

Data from the Arabian Peninsula, including Saudi Arabia, suggested that extended-spectrum and AmpC beta-lactam-resistant bacteria constitute a major problem in nosocomial and community-acquired infections [15,16]. However, there is scarce information on the occurrence and genetic characteristics of β-lactamase-producing bacteria in wild pet animals. Therefore, this study was carried to investigate the occurrence and distribution of beta-lactamase encoding genes within enterobacteria derived from wild pet animals in Saudi Arabia.

## Materials and Methods

### Ethical approval

The fecal samples were collected aseptically with adequate precautionary measures to minimize pain and/or discomfort to the animals and carried out in accordance with the Saudi animal welfare laws.

### Bacterial strains

A total of 17 positive ESBL/AmpC enterobacterial isolates recovered from 75 fecal samples of wild animals at pet market, Taif, Western Saudi Arabia (5 rock hyrax, 4 Yemen Linnet, 3 common kestrel, 3 red foxes, 3 long-tailed finches, 2 caracal, 2 peacock, 1 rock dove, 1 hamadryas baboon, 1 orange-winged parrot, 1 Burmese python, 1 Hill Mynah, 1 African gray parrot, 1 common myna) were included. Wild animals are caught or bought for pet, shops, local breeder or traded (sometimes illegally). The enterobacterial isolates were 9 *E. coli*, and single isolates of *Klebsiella pneumonia*, *Klebsiella oxytoca*, *Proteus* *mirabilis*, *Proteus vulgaris*, *Enterobacter cloacae*, *Enterobacter aerogenes*, *Citrobacter freundii*, and *Citrobacter youngae*. Isolates were identified and confirmed by commercially available biochemical test (API tests; bioMérieux). The ESBLs and AmpC beta-lactamase production were achieved by commercially available Etest (bioMérieux).

### Molecular investigation

Rapid DNA preparation was performed by a boiling technique that includes heating at boiling of an overnight bacterial culture (200 µl) mixed with 800 µl of distilled water, followed by cooling, centrifugation and the supernatant was used as the DNA template for the polymerase chain reaction (PCR).

**Figure-1:**
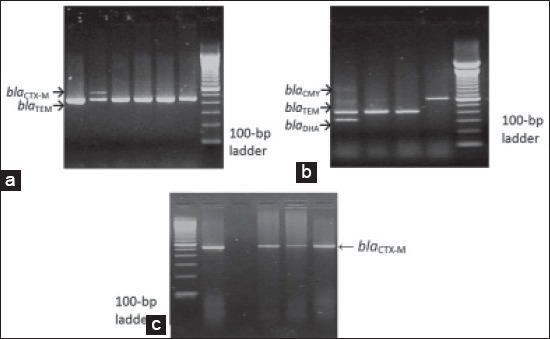
(a) The result of the multiplex polymerase chain reaction (PCR) amplification of the DNA target gene loci of 593-bp fragment DNA region coding for CTX-M; 431-bp fragment DNA region coding for TEM, (b) the result of the multiplex PCR amplification of the DNA target gene loci of: 695-bp fragment DNA region coding for CMY-2; 431-bp fragment DNA region coding for TEM; 314-bp fragment DNA region coding for DHA, (c) the result of the multiplex PCR amplification of the DNA target gene loci of 593-bp fragment DNA region coding for CTX-M.

The presence of genes encoding TEM, SHV, OXA, CTX-M, CMY-2, and DHA type β-lactamases was studied by multiplex PCR using universal primers and conditions previously reported [17,18]. The PCR was conducted in a Thermal Cycler PXE-0.5 (THERMO; Electron Corporation) and the resulting PCR products were subjected to electrophoretic separation in 1.5% agarose gel. Visualization of amplicons was completed by staining with ethidium bromide (Sigma-Aldrich) (1 µg/ml) under UV transluminator and photographed. DNA bands of each amplicon were compared with 100-bp DNA mass marker (Figure-1a-c). Primers sequence and PCR condition are presented in Table-1.

**Table-1:** Primers used in this study to detect betalactamase (*bla*) genes.

Primer target	Primer name	Sequence (5’-3’)	Annealing temperature	Product size (bp)	Reference
TEM (*bla*_TEM_)	TEM-F TEM-R	AGTGCTGCCATAACCATGAGTG CTGACTCCCC GTCGTGTAGATA	61°C for 1 min	431	[18]
SHV (*bla*_SHV_)	SHV-F SHV-R	GATGAACGCTTTCCCATGATG CGCTGTTATCGCTCATGGTAA	61°C for 1 min	214	[18]
CTX-M (*bla*_CTX-M_)	CTX-M-F CTX-M-R	ATGTGCAGYACCAGTAARGTKATGGC TGGGTRAARTARGTSACCAGAAYCAGCGG	61°C for 1 min	593	[17]
OXA (*bla*_OXA_)	OXA-F OXA-R	ACACAATACATATCAACTTCGC AGTGTGTTTAGAATGGTGATC	61°C for 1 min	813	[17]
PampC (*bla*_CMY-2_)	CMY-F2 CMY-R2	AGCGATCCGGTCACGAAATA CCCGTTTTATGCACCCATGA	61°C for 1 min	695	[18]
PampC (*bla*_DHA_)	DHA (F) DHA (R)	GTGGTGGACAGCACCATTAAA CCTGCGGTATAGGTAGCCAGAT	61°C for 1 min	314	[18]

## Results

### PCR detection of ß-lactamase encoding genes

A total of 17 beta-lactamase positive enterobacterial strains recovered from the feces of wild pet animals were screened for beta-lactamase (*bla*)-encoding genes. The PCR screening identified the presence of the beta-lactamase genes encoding TEM, CTX-M, CMY-2, and DHA in 14 of them (Figure-1a-c). None of the isolates were reacted positively for *bla*_OXA_ and *bla*_SHV_. No beta-lactamase genes were identified in the remaining three isolates.

Overall, variety of beta-lactamase genes were found within nine bacterial species isolated from various wild pets species. TEM enzyme was detected in nine isolates of beta-lactamase-producing, respectively, which included 4 isolates of *E. coli* and single isolate of *E. aerogenes*, *P. mirabilis*, *C*. *youngae*, and *P. vulgaris* (Table-2). The CTX-M enzyme was identified in five strains among of beta-lactamase-producing isolates, as a single isolate of *E. coli, K*. *pneumonia*, *E. cloacae*, *K. oxytoca* and *C. freundii* (Table-2). Both of CMY-2 and DHA, a plasmid-mediated AmpC beta-lactamases were detected in two different isolate of *E. coli* (Table-2).

**Table-2:** Prevalence and multiplicity of β-Lactamase genes among ESBLs- positive fecal bacteria derived from wild pet animals in Saudi Arabia.

Bacterial species	ESBL positive no	β-Lactamase- associated genes					

		TEM	CTX-M	CMY-2	CTX-M, TEM	TEM, DHA	Total
*E. coli*	9	3	1	1	-	1	6
*K. pneumonia*	1	-	-	-	1	-	1
*P. mirabilis*	1	1	-	-	-	-	1
*E. cloacae*	1	-	1	-	-	-	1
*K. oxytoca*	1	-	1	-	-	-	1
*C. youngae*	1	1	-	-	-	-	1
*C. freundii*	1	-	1	-	-	-	1
*P. vulgaris*	1	1	-	-	-	-	1
*E. aerogenes*	1	1	-	-	-	-	1
Total	17	7	4	1	1	1	14

*E. aerogenes=Enterobacter aerogenes, K. pneumonia=Klebsiella pneumonia, K. oxytoca=Klebsiella oxytoca, P. mirabilis=Proteus mirabilis, P. vulgaris=Proteus vulgaris, E. cloacae=Enterobacter cloacae, C. freundii=Citrobacter freundii, C. youngae=Citrobacter youngae*, TEM=Temoneira, DHA=Dhahran, CTX-M=Cefotaxime – Munich, CMY=Cephamycinase, SHV=Sulfhydryl Variable, ESBL=Extended spectrum β-Lactamase

### Distribution of bla genes

The ß-lactamase-producing isolates were distributed into two categories, the first harbored only one type of ß-lactamase encoding gene, the second harbored two types (Table-2). Twelve (12/17) of the total beta-lactamase-producing entrerobacteria were harboring only one beta-lactamase encoding gene, including five strains of *E*. *coli* and a single isolate of *E. cloacae*, *K. oxytoca*, *C. youngae*, *P. vulgaris*, *C. freundii*, *P. mirabilis* and *E. aerogenes*.

The *bla*_TEM_, a narrow-spectrum ß-lactamase was detected alone in 7 isolates; *E. coli* (3 isolates) and a single isolate of *C. youngae*, *P. vulgaris*, *P. mirabilis* and *E. aerogenes*. The *bla*_CTX-M,_ an extended-spectrum ß-lactamase was detected alone in four isolates; single isolate of *K*. *oxytoca* from Yemen linnet feces, *E. coli* from common kestrel, *E. cloacae* from rock dove, and *C. freundii* from African gray parrot (Table-3). The plasmid-mediated ß-lactamases, *bla*_CMY-2_ and *bla*_DHA_ were detected in two different *E. coli* isolates recovered from Arabian red fox and Hill Mynah, respectively.

**Table-3:** Genotypic characteristics and occurrence of β-lactamases encoding genes in enterobacteria from wild pet animals.

Isolate ID	Bacteria (no)	Animal species (scientific name)	*bla* gene	Betalactam resistance phenotype
RH-1	*E. coli* (1)	Rock hyrax (*Procavia capensis*)	TEM	AMP, CEP
CK-3	*E. coli* (1)	Common kestrel (*Falco tinnuculus*)	CTX-M	AMP, CEP, AZT, CXM, CTX, CAZ
HM-7	*E. coli* (1)	Hill mynah (*Gracula religosa*)	TEM, DHA	AMC, AMP, CEP, AZT, CXM, CTX, CAZ, FOX
AF-17	*E. coli* (1)	Arabian red fox (*Vulpes vulpes*)	CMY-2	AMC, AMP, CEP, AZT, CXM, CTX, CAZ FEP, FOX
OP-22	*P. mirabilis* (1)	Orange-winged Parrot (*Amazona amazonica*)	TEM	AMP, CEP
BP-19	*C. youngae* (1)	Burmese python (*Python molurus*)	TEM	AMP, CEP
LF-27	*E. aerogene* (1)	Long-tailed finches (*Taeniopygia guttata*)	TEM	AMP, CEP
RD-33	*E. cloacae* (1)	Rock dove (*Columba livia*)	CTX-M	AMP, CEP, CXM, AZT, CAZ
PC-6	*P. vulgaris* (1)	Peacock (*Pavo cristatus*)	TEM	AMP, CEP
BM-11	*K. pneumonia* (1)	Baboon Monkey (*Papio hamadryas*)	TEM, CTX-M	AMP, CEP, CXM, AZT, CAZ
CA-31	*E. coli* (1)	Caracal (*Caracal caracal*)	TEM	AMP, CEP
CM-29	*E. coli* (1)	Common myna (*Acridotheres tristis*)	TEM	AMP, CEP
YL-8	*K. oxytoca* (1)	Yemen linnet (*Carduelis yemenensis*)	CTX-M	AMP, CEP, AZT, CEF, CXM, CAZ
AP-13	*C. freundii* (1)	African gray parrot (*Psittacus erithacus*)	CTX-M	AMP, CEP, AZT, CXM, CTX, CAZ

E. aerogenes=Enterobacter aerogenes, K. pneumonia=Klebsiella pneumonia, K. oxytoca=Klebsiella oxytoca, P. mirabilis=Proteus mirabilis, P. vulgaris=Proteus vulgaris, E. cloacae=Enterobacter cloacae, C. freundii=Citrobacter freundii, C. youngae=Citrobacter youngae

A total of two (2/17) of the total beta-lactamase-producing isolates were harboring gene combinations of *bla*_TEM_ and *bla*_DHA_ in *E. coli* recovered from the feces of Hill Mynah and *bla*_TEM_ and *bla*_CTX-M_ in *K. pneumonia* delivered from the feces of baboon monkey.

## Discussion

The resistance to beta-lactam and beta-lactamase inhibitors is of great clinical significance in several countries. Resistance to beta-lactam antibiotics is primarily mediated by beta-lactamases production. Many different β-lactamases have been described, but TEM, SHV, OXA, CMY-2, and CTX-M β-lactamases are currently regarded the most common among *Enterobacteriaceae* spp. [2].

Recently, many studies carried out in different countries describing the prevalence and characteristics of beta-lactamase gene harbored *Enterobacteriaceae* in wildlife free-living Canada geese in Georgia and North California [19], wild animals in Portugal [8,20], zoo animals in Japan [21], black-headed gulls in the Czech Republic [4] and wild birds and free-range poultry in Bangladesh [22]. Since there seem to be geographical variations in the occurrence of different ESBLs, we describe prevalence and characteristics of ESBL/AmpC-genotypes within enterobacterial isolates from wild pet animals presenting at live animal market in Taif, Western Saudi Arabia.

### Prevalence of beta-lactamase genes

The beta-lactamase genes harboring enterobacterial isolates from wild pet animals were detected in 14 out of 17 isolates including six *E. coli* and single isolate of *K. pneumonia*, *P*. *mirabilis*, *E. cloacae*, *K. oxytoca*, *C. youngae*, *C. freubdii, P. vulgaris*, and *E. aerogenes*. The rate of *bla* genes in this study was consistent with that previously reported [8,20,21], whereas *E. coli* is the most prevalent and encountered *bla* genes among enterobacteria from wild animals.

### Determination of the types of bla genes

In this study, PCR screening revealed detection of beta-lactamase encoding genes of TEM, CTX-M, CMY, and DHA. None of the isolates were positive for *bla*_OXA_ and *bla*_SHV_. The remaining three isolates did not show any of the *bla* genes investigated. Similarly, previous studies also detected many β-lactamase-encoding genes in wild animals [12,14,20,21,22].

A TEM-β-lactamase is a narrow-spectrum beta-lactamase gene, which confers resistance against penicillin’s and first-generation cephalosporins [23]. In this study, *bla*_TEM_ being detected in 7 isolates out of 17 enterobacteria-producing beta-lactamase as a sole mechanism of resistance to beta-lactams and all these isolates showed an ampicillin, cephalothin and or cefuroxime resistance phenotypes. TEM-β-lactamase has been previously detected in fecal isolates from magpies and wild rabbits from West Wales [24], free-living Canada geese in Georgia and North Carolina [19], wild animals in Portugal [20], Zoo animals in Japan [21], black-headed gulls in the Czech Republic [4], yellow-legged gulls in France [5], imported flamingos in Japan [25], gulls population in Sweden [12], migratory and resident population of rooks in Austria [26], seagulls and crows in Bangladesh [27].

Recently, there has been worldwide increase in the incidence of ESBLs [3]. In this study, *bla*_CTX-M_, an ESBL-encoding gene, was detected in five isolates of enterobacteria from feces of wild animals. The *bla*_CTX-M_ has been previously identified in fecal bacteria from wild animals in Portugal [20], masked palm civet in Japan [21], imported flamingos in Japan [25], gulls in Sweden [12], migrating and resident population of rooks in Austria [26].

Furthermore, the plasmid-mediated AmpC genes (*bla*_CMY-2_ and *bla*_DHA_), were observed in two of strains of enterobacteria showed a typical AmpC-beta-lactamase resistance phenotype. The presence of AmpC β-lactamases have been found worldwide but are less common than ESBLs [28]. The information on the presence of AmpC producing *Enterobacteriaceae* in wildlife is scarce. Recently, the *bla*_CMY_ has been reported previously from jaybird isolates of *K*. *oxytoca* in Japan [21], migrating and resident population of rooks in Austria [26]. The *bla*_DHA_ was the first identified from clinical isolates of *Salmonella enteritidis* in Saudi Arabia [29]. Recently, in Magnolia, the *bla*_DHA_ was detected in one *E. coli* from clinical sources [30].

### Analysis of bla genes multiplicity among isolates

As in previous studies, bla-genes in this study were detected within enterobacteria from wild animals either as a single gene loci or as gene combination of two or more gene loci for beta-lactamases [13,21,26].

A comparative view of Arabian Gulf region and Saudi Arabia showed a high occurrence of ESBL-producing isolates harboring TEM, SHV, OXA, and CTX-M- β-lactamases from hospitals [16,31-34] and raw chicken [35].

## Conclusions

It is of interest the detection of ESBL/AmpC-producing bacteria in wild animals at pet market. This is the first study, to our knowledge, of enterobacteria harboring β-lactamase genes in wild animals in Saudi Arabia. The fact that these animals often live in close contact with their owners and other people in market make the occurrence of transmission between them even more likely. More studies should be carried out in the future in order to track the variants and evolution of β-lactamase genes compared to those from human isolates.

## Authors’ Contributions

SAH conceived, designed the study, drafted and revised the manuscript. MYS collected and analyzed samples. Both authors read and approved the final manuscript.
